# Evaluating the Impact of Post-Emergence Weed Control in Honeybee Colonies Located in Different Agricultural Surroundings

**DOI:** 10.3390/insects12020163

**Published:** 2021-02-14

**Authors:** Ivana N. Macri, Diego E. Vázquez, Eduardo A. Pagano, Jorge A. Zavala, Walter M. Farina

**Affiliations:** 1 Laboratorio de Insectos Sociales, Departamento de Biodiversidad y Biología Experimental, Facultad de Ciencias Exactas y Naturales, 1428 Buenos Aires, Argentina; macri.ivana@inta.gob.ar (I.N.M.); diegovazquez@bg.fcen.uba.ar (D.E.V.); 2 Instituto de Fisiología, Biología Molecular y Neurociencias (IFIBYNE), CONICET-Universidad de Buenos Aires, 1428 Buenos Aires, Argentina; 3 Instituto de Ingeniería Rural, Centro de Investigación de Agroindustria (CIA), Instituto Nacional de Tecnología Agropecuaria (INTA), Hurlingham, 1686 Buenos Aires, Argentina; 4 Cátedra de Bioquímica, Facultad de Agronomía, Universidad de Buenos Aires, 1428 Buenos Aires, Argentina; epagano@agro.uba.ar (E.A.P.); zavala@agro.uba.ar (J.A.Z.); 5 Instituto de Investigaciones en Biociencias Agrícolas y Ambientales (INBA), CONICET-Universidad de Buenos Aires, 1417 Buenos Aires, Argentina

**Keywords:** *Apis mellifera*, agricultural intensification, herbicides, *cytochrome P450*, pesticides detoxification

## Abstract

**Simple Summary:**

The honeybee *Apis mellifera* is one of the main pollinators in agricultural ecosystems and therefore they are exposed to pesticides and the reduction of floral diversity. *Cytochrome P450 monooxygenases* are enzymes involved in xenobiotic detoxification used by organisms, including insects. In this study, we evaluated honeybee foraging activity and the expression profiles of several cytochromes, before and after the administration of a mixture of three of the most used herbicides in the region during the summer season. Additionally, we investigated whether colonies located in three distinct agricultural environments with different crop/wild flora proportions are affected similarly or differently by herbicide administration. We found that the expression of several *cytochrome P450* genes decreased significantly in larvae after post-emergence weed control and they showed significant differences between apiaries in the case of honeybee workers. Besides, we found significant positive correlations between pollen collection and some pesticide detoxification genes. Our results support that diversity and quality of resource availability as well as the presence of herbicides affect colonies’ nutritional state and bee health. Moreover, their detoxification response shows that larvae are more harmed than adults in these agricultural ecosystems.

**Abstract:**

The honeybee *Apis mellifera* is exposed to agricultural intensification, which leads to an improved reliance upon pesticide use and the reduction of floral diversity. In the present study, we assess the changes in the colony activity and the expression profile of genes involved in xenobiotic detoxification in larvae and adult honeybees from three apiaries located in agricultural environments that differ in their proportion of the crop/wild flora. We evaluated these variables before and after the administration of a mixture of three herbicides during the summer season. The expression of several *cytochrome P450 monooxygenases* decreased significantly in larvae after post-emergence weed control and showed significant differences between apiaries in the case of honeybee workers. Principal component analysis (PCA) revealed that colonies located in the plot near to a wetland area exhibited a different relative gene expression profile after herbicide application compared with the other plots. Moreover, we found significant positive correlations between pollen collection and the pesticide detoxification genes that discriminated between plots in the PCA. Our results suggest that nutrition may modify herbicide impact on honeybees and that larvae are more harmed than adults in agroecosystems, a factor that will alter the colonies’ population growth at the end of the blooming period.

## 1. Introduction

Honeybees (*Apis mellifera* L.) are one of the main pollinators in natural and agricultural ecosystems [1]; however, their populations are growing at a slower pace than the demands for pollination services [2,3]. The declines of beehives in many countries have been attributed to multiple factors, including pathogens, parasites, habitat loss and fragmentation [4,5]. In addition to these factors, bees are exposed to agrochemicals in agricultural landscapes and intensive mono-cropping systems which lead to reduced floral resources and nutrition [4,6]. Managed colonies used for pollination services face a less diversified diet of pollens, which might not provide all essential nutrients [7]. Planting buffer zones at field borders has been tested and recommended in several countries to reduce the drift of pesticides into adjacent habitats [8]. However, since the buffer zone itself often contains flowers that attract pollinators, an additional in-field buffer zone can be used to protect pollinators from drifting pesticides and to offer them diverse food sources [9]. In this sense, honeybees depend on the adequate availability and collection of pollen to meet most of their dietary needs. Stress and exposure to pesticide mixtures decrease pollen foraging performance in honeybees [10,11], leading to a nutritional imbalance with a pollen deficit at the colony level, and thereby affects colony development [10]. Hence, pollen balance is central to the growth and sustainability of colonies affecting many downstream processes such as brood rearing and behavioral development of workers, and also interactions between diet, nutrition and disease and/or immune system status [12].

Latin American countries use genetically modified (GM) crops extensively and have become increasingly dependent on herbicides, mainly glyphosate, for weed control, especially in no-till production, as in the case of Argentina and Brazil [13]. In Argentina, no-tillage agriculture is the most widely used crop system, occupying 90% of the surface [14]. This model depends exclusively on the application of herbicides as the only form of weed control and the most used are chemical formulations based on these active ingredients: glyphosate (GLY), 2,4-dichlorophenoxyacetic acid (2,4-D) and atrazine [15]. On one hand, GLY is a broad-spectrum herbicide which inhibits the enzyme 5-enolpyruvylshikimate-3-phosphate synthase (EPSPS), part of the shikimate pathway, in higher plants, algae and bacteria [16]. On the other hand, 2,4-D is an organic and systemic herbicide which acts by mimicking the action of the plant growth hormone auxin, which results in uncontrolled growth and eventually the death of susceptible plants [17]. Lastly, atrazine is a triazine class herbicide which acts as a selective herbicide that inhibits photosynthesis in susceptible plants [18]. According to these practices, it is important to mention the good agricultural practice guidelines for agrochemical application in Argentina. For instance, agrochemical products must be used only for authorized crops, at the prescribed doses and waiting periods, and following all the safety information on the label [19]. This reduces the possibility of drift to non-target vegetation. It is also recommended to have the respective “Safety Data Sheets” of the agrochemicals used [19].

Honeybee foragers make several flights per day to gather resources and, in doing so, agrochemicals which might be present in the flowers visited after spray applications may also be circulated among hive mates and present in the stored resources [20]. Substances that are taken into the hive can remain stored for long periods and accumulate until the resources are used as supplies for the colony [21]. GLY and other pesticides are not an obstacle for bees collecting floral nectar that contains it [22]. Indeed, bees display a preference for flowers that contain GLY in sugar water at 10 ppb [23]. However, GLY exposure impairs cognitive abilities that could impact foraging efficiency and in-hive coordination of collective activities [24,25,26]. Inside their colonies, after bees collect food from contaminated sources, this herbicide could become concentrated because nectar is evaporated and condensed by food processor bees to make honey and bee bread [27]. The impact of sub-lethal chronic effects is particularly important for social insects since they could affect the entire bee colony [20]. The stored pollen is composed of a mixture of different plants present in variable amounts, reflecting the floral composition of the environment of the hive [28]. In areas of extensive cultures of polleniferous crops, large amounts of pollen from crops enter the colony [28]. Beebread is mainly composed of pollen, which is consistently contaminated by pesticides [29]. Bees add honey and bee secretions to the pollen to make a nutritional protein source for adult and developing bees, with large amounts being consumed by nurse bees, and to a lesser extent by larvae [28]. Traces of GLY and 2,4-D were found in honey samples [30,31,32,33]. Meanwhile, atrazine was one of the most frequently found residues in pollen, wax and bees from North American apiaries, including brood combs where bees lay eggs and larvae develop [34].

Realistic field exposures are complex to carry out experimentally because there are many concomitant factors during the pesticide administration, as the effect of the combination of different substances (a mixture of agrochemical formulations) and low concentrations of residues mainly detectable close to the administration day [9,35]. Hence, measurements of the internal physiological state of animals with biomarkers of response are recommended in open field assessments to reveal signs of stress without conspicuous symptoms [36]. At the molecular level, exposure to pesticides can activate detoxification pathways in honeybees [36,37,38,39,40,41] and modulate the expression of genes involved in immunity and behavioral maturation [40,42]. The herbicides GLY and atrazine promoted lipid peroxidation [43] and altered acetylcholinesterase activity [44] and the carotenoid–retinoid system in honeybees [45]. *Cytochrome P450 monooxygenases* (*P450s*) are among the principal phase I detoxification enzymes used by organisms, including insects, to metabolize xenobiotics, including phytochemicals and insecticides [46]. Despite this dependence on *P450s*, with 46 genes, the honeybee genome is reduced even in comparison with some other hymenopterans [47]. The diminished repertoire of detoxifying genes in the honeybee might stem from compensatory mechanisms associated with their highly social behavior, including social immunity [48,49] and a social detoxification system, which focuses on how behavioral dynamics of the colony can reduce the burden of toxic substances on the detoxification system of individual members [47]. Some research suggests that expression profiling can be used to identify stressor-specific biomarkers in bees because they have distinct genetic pathways for dealing with pathogenic, nutritional and xenobiotic challenges that are associated with specific and thus diagnostic changes in gene expression [50]. Accordingly, the honeybee becomes a suitable sentinel species for pollinator community, especially those individuals undergoing development within beehives, given that they are much more vulnerable to environmental challenges [20].

In this study, we aimed to evaluate the medium-term impact of post-emergence weed control on the detoxification pathways and foraging activity of honeybee colonies located in crop settings following the good agricultural practice guidelines for agrochemical application in Argentina [19]. Furthermore, we analyzed if this impact depends on the characteristics of the crop and buffer zones with wild flora around beehives.

## 2. Materials and Methods

### 2.1. Study Site and Animals

The behavioral study and sample collection were performed during the summer season, in hives of European honeybees (*Apis mellifera* L.) located on a farm near Carlos Casares in the humid Pampean region of Argentina (35°57′7.29″ S, 61°13′6.33″ O). Within the farm, different crops such as soybean (*Glycine max*), sunflower (*Helianthus annuus*) and corn (*Zea mays*) were sown. Sunflower (Syngenta 3950), soybean (Nidera 4611 and Don Mario 4615) and corn (Dekalb 73-10 and GL Stack 4500) were genetically modified crops tolerant to herbicides. Soybean and corn are visited by bees searching for nectar or pollen, respectively [51,52], while sunflower is a melliferous crop commonly visited by bees searching for nectar and pollen [28,52]. The soybean crop was sown at the end of November 2016 (second planting) with seeds with a short development cycle, while the sunflower and corn crops were sown in mid-October. In all cases, the agricultural settings were treated with pre-planting weed control herbicides during spring using a land sprayer (3 L/ha) before beehives arrived at the site. Standard beekeeping practices were applied, and beehives did not show observable signs of disease during the experiment.

The post-emergence weed control on the different crops was done in early January 2017 with a land sprayer (1.5 L/ha). The first sampling (in mid-December 2016) was done 20 days before this treatment, while the second one (at the end of January 2017) was done 20 days after treatment. The weed control was performed with a mixture of 3 commercially formulated herbicides: an atrazine-based herbicide (Gesaprim^®^ Syngenta), a 2,4-D-based herbicide (Voleris^®^ Syngenta) and a GLY-based herbicide (Sulfosato Touchdown^®^ Syngenta). Treatments with fungicides or insecticides in the crops were not performed during the experiment and nor were beehives treated with antibiotics or acaricides. The three crops were in their vegetation stage at the moment of the first sampling and during the herbicide application, without available flowers for bees [53,54,55]. In contrast, at the moment of the second sampling, all crops were in full bloom. Weather conditions were similar in the two sampling moments and optimal for the foraging activity of bees, with a daily average temperature of 31 and 32 °C and a daily average RH of 32 and 30%, respectively. Furthermore, both sampling dates were sunny days without wind nor rainfall. This period matches the peak of nectar flow in the region and consequently the maximum population size of honeybee colonies.

**Figure 1:**
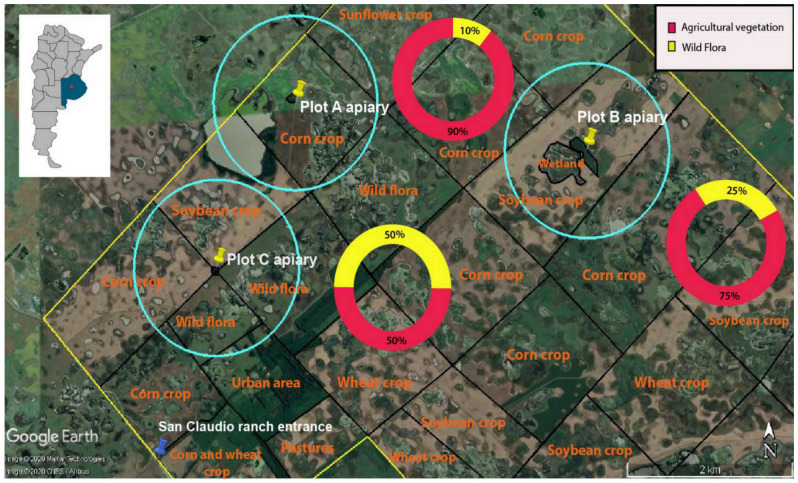
Geographical location of the three studied apiaries on San Claudio Farm, Province of Buenos Aires, Argentina. Light blue circles show 1 km radius around the apiary, indicating the potential foraging area. Circles with a color classification indicate the percentage of cultivated area (in orange, type of crop/vegetation) and in yellow the percentage of uncultivated area with wild flora in each plot (details in Materials and Methods).

Three crop settings or plots were chosen according to the presence of an apiary in their surroundings (Figure 1). In all cases, beehives of each plot were separated by at least 2.5 km. According to previous studies, an average honeybee colony conducts much of its foraging behavior in agricultural settings within several hundred meters of the hive, while in forest areas, this behavior shows a modal, median and mean distance of 0.7, 1.6 and 2.2 km around, respectively [27]. Thus, overlapping of the foraging areas of colonies located at different apiaries was unlikely. The plots differed from each other in the type and proportion of crop and uncultivated area with wild flora, with most of them being melliferous vegetation, such as *Diplotaxis tenuifolia*, *Conyza bonariensis*, wild *Cucurbita* and different species of thistle, dandelion, eucalyptus, clover and locust trees. The different species were collected in a herbarium and subsequently identified with a taxonomic key of the native flora of Buenos Aires Province. In increasing order of wild flora proportion, the first apiary, containing 23 hives, was located in a forest patch in plot A. This plot had an area of 189 ha and it was mainly seeded with corn and was placed 50 m from a sunflower crop. Furthermore, in addition to corn, the field was seeded with soybean and this plot had a low percentage of wild flora in the surroundings (10%). The percentage of wild flora was estimated with a satellite image of the farm in which we calculated the crop area within the foraging area of each apiary (circumferences of 2 km in diameter). The second apiary, containing 33 hives, was located in a forest patch with a wetland area in plot B. This plot had an area of 189 ha and it was mainly seeded with soybean and surrounded by corn. The wetland area provided a diversity of flowers that were not present in the other apiaries, such as different species of the genera *Thypa*, *Eryngium* and *Juncus*. In sum, the proportion of wild flora, in this case, was 25%. The third apiary, containing 44 hives, was situated in a forest patch in plot C. This plot had an area of 178 ha and it was 20 m from soybean and corn crops on one side and wild flora on the other side, in a proportion of 50% each. Wild flora in the uncultivated area was the only source of food for bees during the first sampling in all plots.

### 2.2. Colony Activity

For the behavioral study, in each sampling moment, we recorded the number of incoming bees (incoming rate) at the entrance of the hives as an indicator of the colony activity and a biomarker of the effect of the herbicide mixture application. These foragers were individuals of different ages but in the second sampling, in all cases, they started to visit flowers outside the hive after the post-emergence weed control in the neighboring crops. Bear in mind the average developmental time of honeybees in their different stages, 19–20 days for the pre-imaginal stage, around 21 days of behavioral development of adult bees inside the hive and two or three weeks of foraging behavior during summer [27].

In each apiary, we randomly chose eight colonies with similar population sizes formed by a mated queen, three or four brood frames and food reserves. The eight randomly selected hives represented 18–35% of each apiary, being representative of the colonies in each plot. We counted the arrivals in the eight chosen hives for 1 min per day, from 13 to 16 h, within the bees’ most active hours [27]. According to the presence or absence of pollen loads on their hind legs, incoming bees were recorded as pollen or non-pollen bees, respectively. These recordings were carried out in both sampling moments in the same hives, before (20 December 2016) and after (30 January 2017) the post-emergence weed control performed outside in the neighboring crops. Furthermore, a ratio between pollen foragers and the total incoming rate was calculated in each sampling moment.

### 2.3. Sample Collection

To assess the internal physiological state of bees inside the hives without direct exposure to the external agricultural environment, we sampled bees and larvae from four of the eight randomly selected colonies in each of the apiaries [35,36]. In each colony, we captured bees that were located in the brood area of the hive (henceforth: hive bees) for subsequent in situ gut dissection and also sampled five larvae in the fifth instar of the same brood frame. Larvae were pooled in situ in a single cryovial that was immediately placed into liquid nitrogen until RNA extraction. Then, once we had sampled hive bees and larvae from all selected colonies in each plot, the hive bees were anesthetized for four min at −20 °C. Therefore, ten midguts for each of the four colonies were dissected and pooled in a single cryovial and conserved in liquid nitrogen until RNA extraction. Thus, we utilized four biological replicates per apiary.

These sample collections were carried out in both sampling moments in the same hives, before (20 December 2016) and after (30 January 2017) the post-emergence weed control performed outside in the neighboring crops. On one hand, we sampled larvae in the fifth instar due to their diet, as it is the larval instar that is mainly exposed to the resources collected in the external environment (honey and beebread derived from nectar and pollen) [35]. In the second sampling, these larvae hatched from their eggs fifteen days after weed control. On the other hand, we captured bees located in the brood area because they are young workers which concentrate on tasks such as processing food, cleaning cells, feeding brood and tending the queen [27]. Thus, these bees come into contact with the food brought into the colony yet have no direct interaction with the outside environment. In the second sampling, most of these young workers had emerged after the weed control.

### 2.4. Gene Expression Analysis

The internal physiological state of hives bees and larvae was assessed with a set of molecular biomarkers of the detoxifying and immune response. Changes in the transcription of this gene set are signs of chemical stress associated with the intake of herbicides in food [36,41,42].

Total RNA was extracted from each set of pooled midguts and larvae with a TRIzol extraction protocol (Invitrogen Life Technologies, Waltham, MA, USA). Total RNA was treated with DNase, before reverse transcription (RT) reactions. The cDNA was synthesized (Revertaid RT, Thermo Fisher Scientific, Waltham, MA, USA) with an input of 1 µg of total RNA.

The qRT-PCR reactions were performed using three technical replicates for each biological replicate to assess the expression level of antimicrobial peptide *Abaecin* (*AB*) and *P450 cytochromes CYP6AS2*, *CYP6AS3*, *CYP6AS4*, *CYP6BD1* and *CYP9Q3*, involved in phase I of the xenobiotic detoxification pathways in honeybees [47], using previously validated primers (Table 1). The comparative qRT-PCR analysis was performed using the Applied Biosystems 7500 software [56] following the manufacturer’s protocol for the ∆∆Ct method. For the correct use of this method, the efficiencies of the primers for both targets and housekeeping were calculated and validated (Table S1). Melting curve analysis was used to ensure amplification specificity. *Rpl8* was used as the endogenous control [57,58] and samples of the first sampling moment were taken as a control to compare how different the relative expression of our selected targets after the post-emergence weed control was.

### 2.5. Statistics

All statistical tests were performed with R v3.5.1 [59]. Colony activity rates and gene expression data from real-time qRT-PCR were analyzed with a paired Wilcoxon rank test for comparison between sampling moments and with an alpha level of 0.05. A Friedman rank sum test was used for comparison between plots. A Conover post hoc test was used for pairwise comparisons between plots and *p* value corrected with a Bonferroni procedure. All non-parametric test were carried out using the “PMCMRplus” package [60]. Principal component analysis (PCA) and Kendall’s rank test for correlations between the variables studied were performed using the R libraries “ade4” and “psych”, respectively. Furthermore, the “FactoMineR” package [61] was used for PCA graphics.

## 3. Results

### 3.1. Colony Activity

**Figure 2:**
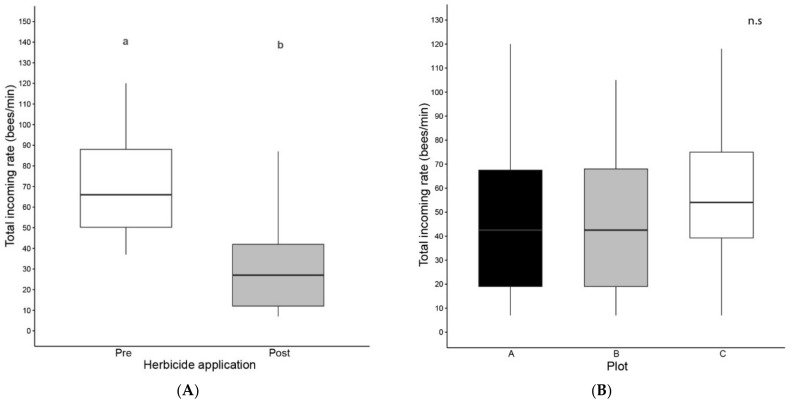

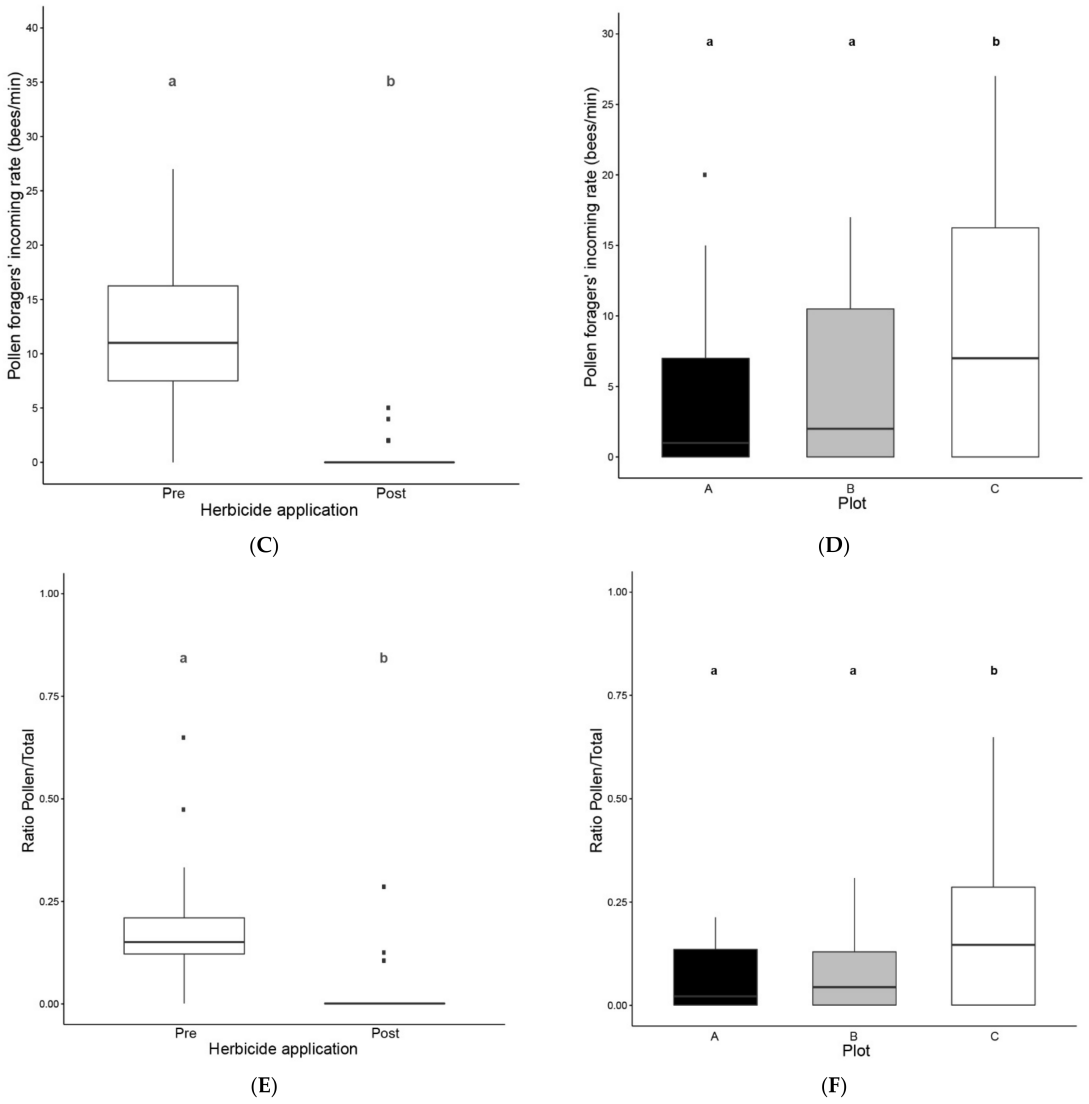
Colony activity. (**A**,**C**,**E**) Before and after post-emergence weed control (**B**,**D**,**F**) for each plot. (**A**,**B**) Number of bees entering the hive per minute, (**C**,**D**) number of bees entering the hive with pollen baskets per minute and (**E**,**F**) ratio of bees entering the hive with pollen baskets with regard to the total number of bees entering the hive. Boxplot shows the median and interquartile range (IQR), with whiskers showing the maximum value within 1.5 IQR, and individual points mark values outside this range. No significant differences (n.s.) were found between plots for the number of bees entering the hive per minute (N = 24, Friedman rank sum test). Different letters indicate significant differences between sampling moments (*p* < 0.001, N = 24, Wilcoxon paired test). Different letters indicate significant differences between plots (*p* < 0.001, N = 24, Friedman rank sum test). n.s., not significant.

To estimate the colony activity before and after post-emergence weed control, we evaluated the rate of total incoming bees (bees/min) and pollen foragers (bees with pollen loads/min) in all three apiaries studied. Moreover, we calculated the proportion between pollen foragers and total incoming rate. The three variables differed between sampling moments, before and after post-emergence weed control (Figure 2). The statistical analysis revealed that total incoming rate, pollen foragers’ incoming rate and ratio of pollen/total incoming rate were significantly lower after herbicide administration (Wilcoxon paired test, N = 24, *p* < 0.001, Table S2), even though the availability of floral resources was increased in the second sampling moment. Furthermore, pollen foragers’ incoming rate and ratio of pollen/total incoming rate showed significant differences between plots (Figure 2), with the collection of pollen and the proportion of pollen/total incoming rate being higher in the C plot apiary (Friedman rank sum test, N = 24, *p* < 0.001, Table S2). No significant differences between plots were found in the case of total incoming rate.

### 3.2. Relative Gene Expression

**Figure 3:**
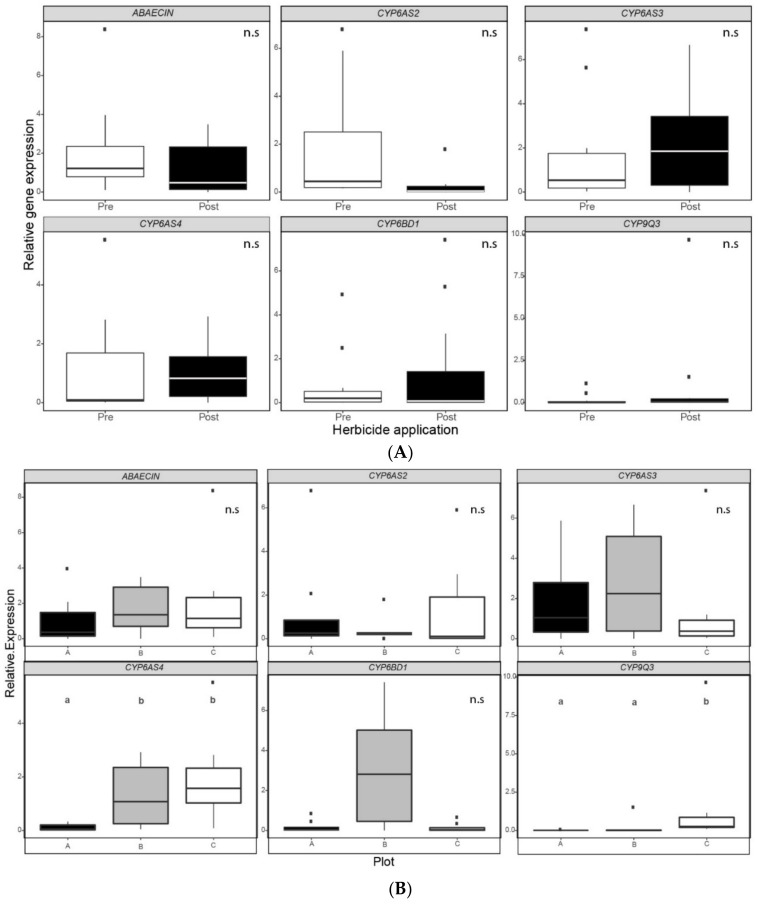
Gene expression in hive bees’ midguts (**A**) before and after post-emergence weed control and (**B**) for each plot. Relative expression levels of the antimicrobial peptide *Abaecin (AB)* and *P450 cytochromes* (*CYP6AS2*, *CYP6AS3*, *CYP6AS4*, *CYP6BD1* and *CYP9Q3*) in ten pooled midguts of hive bees captured inside randomly selected colonies. Transcription was assessed by real-time quantitative reverse transcription amplification (qRT-PCR). Data are presented as a fold change expression normalized to the endogenous reference gene *Rpl8*, calculated by ∆∆CT method. Boxplots show the median and interquartile range (IQR), with whiskers showing the maximum value within 1.5 IQR, and individual points mark values outside this range. Different letters indicate significant differences between plots (*p* < 0.001, N = 12, Friedman rank sum test). No significant differences (n.s.) were found between application moments for any of the genes studied (N = 12, Wilcoxon paired test).

**Figure 4:**
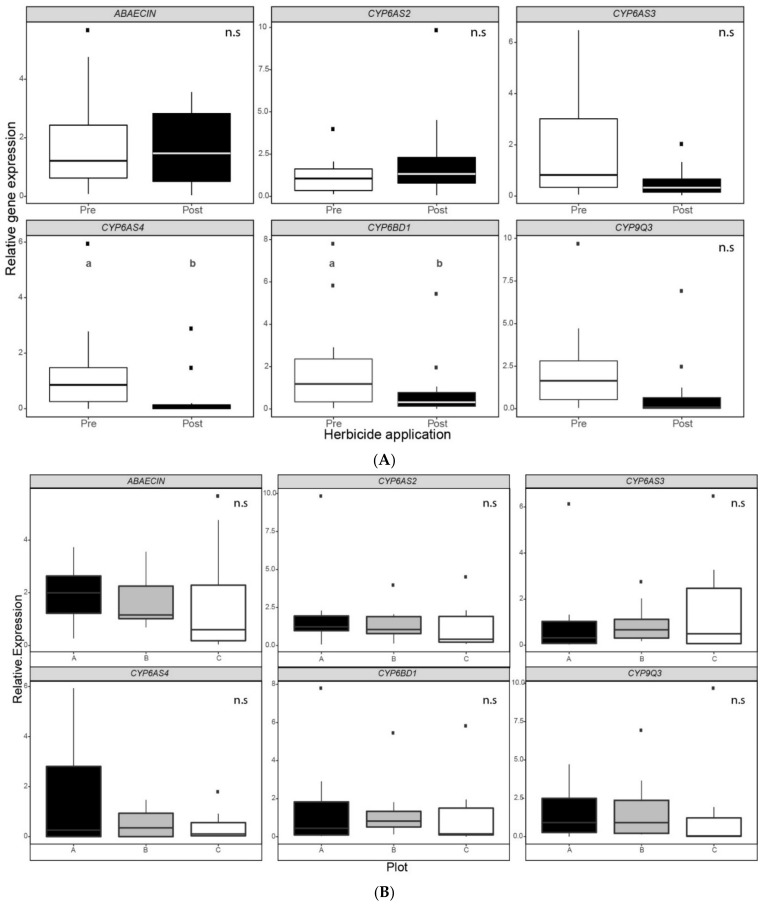
Gene expression in larvae, (**A**) before and after post-emergence weed control and (**B**) for each plot. Relative expression levels of the antimicrobial peptide *Abaecin (AB)* and *P450 cytochromes (CYP6AS2*, *CYP6AS3*, *CYP6AS4*, *CYP6BD1* and *CYP9Q3*) in five polled fifth instars sampled from randomly selected colonies. Relative gene expression was assessed by real-time quantitative reverse transcription amplification (qRT-PCR). Data are presented as a fold change expression normalized to the endogenous reference gene *Rpl8*, calculated by ∆∆CT method. Boxplots show the median and interquartile range (IQR), with whiskers showing the maximum value within 1.5 IQR, and individual points mark values outside this range. Different letters indicate significant differences between application moments (*p* < 0.05, N = 12, Wilcoxon paired test). No significant differences (n.s.) were found between plots for any of the genes studied (N = 12, Friedman rank sum test).

The relative expression levels of antimicrobial peptide *Abaecin (AB)* and *P450 cytochromes CYP6AS2*, *CYP6AS3*, *CYP6AS4*, *CYP6BD1* and *CYP9Q3* were measured to assess the colonies’ physiological state in each plot and for the two sampling moments. This was done for hive bees’ midgut and the whole body of fifth instar larvae. In the case of hive bees’ midguts, no significant differences were recorded between sampling moments (Figure 3). On the contrary, we found significant differences between moments in the relative expression of *CYP6AS4* (Wilcoxon paired test, N = 12, *p* = 0.012) and *CYP6BD1* (Wilcoxon paired test, N = 12, *p* = 0.034) for larvae (Figure 4). In both *P450 cytochromes*, the relative expression was lower after post-emergence weed control (Table S3). However, in the case of larvae, no significant differences were recorded between plots (Figure 3). In contrast, we found significant differences between plots in the relative expression of *CYP6AS4* and *CYP9Q3* (Figure 3). Hive bees’ *P450 cytochrome CYP6AS4* showed a significantly lower relative expression in the apiary of plot A than the apiaries of plots B and C (Friedman rank sum test, N = 12, *p* < 0.001, Table S3). Additionally, hive bees’ *P450 cytochrome CYP9Q3* showed a significantly higher relative expression in the apiary of plot C than the apiaries of plots A and B (Friedman rank sum test, N = 12, *p* < 0.001, Table S3).

### 3.3. Correlation between Variables Studied

Kendall’s rank correlation test for non-normally distributed data shows a strong positive correlation between hive bees’ *P450 cytochrome CYP6BD1* and *CYP6AS2* (tau = 0.779, Z = 3.506, *p* < 0.001), *CYP6AS4* (tau = 0.657, Z = 2.956, *p* = 0.003) and *CYP9Q3* (tau = 0.748, Z = 3.368, *p* < 0.001) relative expression after post-emergence weed control (Table S4). In addition, *CYP6AS2* is significantly and positively correlated with *CYP6AS4* (tau = 0.515, Z = 2.501, *p* = 0.021) and *CYP9Q3* (tau = 0.667, Z = 3.155, *p* = 0.002). Finally, *CYP6AS4* and *CYP9Q3* are significantly and positively correlated as well (tau = 0.848, Z = 4.132, *p* < 0.001). In the case of larvae (Table S5), only *CYP6AS3* and *CYP6AS4* show a significant and positive correlation (tau = 0.461, Z = 2, *p* = 0.046). Correlations between relative expressions of the same biomarker gene for hive bees and larvae were also evaluated (Table S6), being significantly and positively correlated only the *P450 cytochrome CYP9Q3* (tau = 0.504, Z = 2.268, *p* = 0.023). When we analyzed the correlation between pollen collection and relative gene expression for hive bees (Table S7), we found significant and positive correlations between pollen foragers’ incoming rate and either *CYP6AS2* (tau = 0.328, Z = 2.121, *p* = 0.034) or *CYP6AS4* (tau = 0.349, Z = 2.251, *p* = 0.024) relative expression. Furthermore, the ratio of pollen/total incoming rate shows a significant and positive weak correlation with antimicrobial peptide *Abaecin* (tau = 0.369, Z = 2.402, *p* = 0.016).

### 3.4. Principal Component Analysis (PCA)

**Figure 5:**
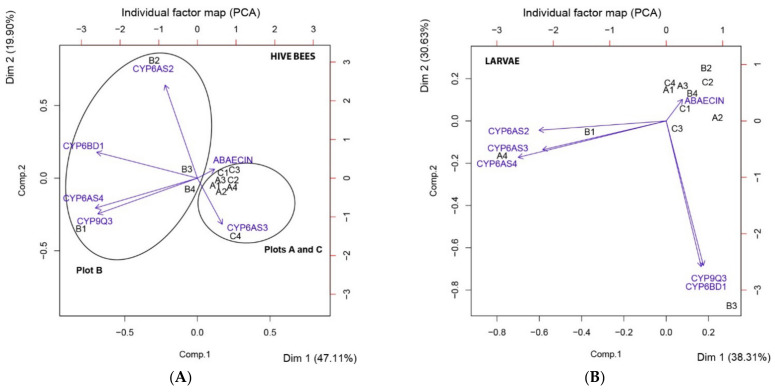
Principal component analysis (PCA) of relative gene expression after post-emergence weed control. (**A**) Individual factor maps (observation level) for relative gene expression of hive bees’ midguts after post-emergence weed control. Variables are expressed in Comp. 1 and 2 (see Table S9). Individual factor map shows the differentiation of plot B from plots A and C, by components 1 and 2, delimited with ellipses. Axes 1 and 2 absorbed 47.10% and 19.90% of the dataset variability, respectively. Each sample is labeled with a letter and a number which indicate plot and hive, respectively. (**B**) Individual factor maps for relative gene expression of larvae after post-emergence weed control. Variables are expressed in Comp. 1 and 2 (see Table S9). Individual factor map does not show any differentiation between plots. Axes 1 and 2 absorbed 38.31% and 30.63% of the dataset variability, respectively. Each sample is labeled with a letter and a number which indicate plot and hive, respectively.

We analyzed the principal components of the relative gene expression variables after the post-emergence weed control for larvae and hive bees separately. On one hand, the three first principal components for the hive bee analysis explain 85.60% of the cumulative proportion of variability (Table S8). *P450 cytochromes CYP6AS4*, *CYP9Q3* and *CYP6BD1* are the set of genes that better explain the first principal component (PC1), while *CYP6AS2* better explains the second principal component (PC2). Thirdly, the antimicrobial peptide *Abaecin* better explains the third principal component (PC3) (Table S9). The dimension limited by PC1 and PC2 discriminates between the individual observations in two groups, plot B on one side and plots A and C on the other side. These two principal components absorb 47.10% and 19.90% of the dataset variability, respectively (Figure 5). Thus, relative expressions of hive bees’ *P450 cytochromes CYP6AS2, CYP6AS4, CYP9Q3* and *CYP6BD1* after the post-emergence weed control are the variables that differentiated the physiological state of hive bees between plots. On the other hand, the three first principal components for larva analysis explain 85.50% of the cumulative proportion of variability (Table S8). *P450 cytochrome CYP6AS4* is the gene which better explains the first principal component (PC1), while *CYP9Q3* and *CYP6BD1* are the genes that better explain the second principal component (PC2). Thirdly, the antimicrobial peptide *Abaecin* better explains the third principal component (PC3) (Table S9). Nevertheless, the principal component analysis does not show any discrimination between plots for the samples (Figure 5).

## 4. Discussion

Our results show a decrease in the three variables of colony activity studied (total incoming rate, pollen foragers’ incoming rate and ratio of pollen/total incoming rate) and in the relative expression level of several stress biomarker genes in larval tissue of all colonies, 20 days after post-emergence weed control in the crops surrounding the beehives. However, no global significant differences between sampling moments were found in the case of the relative expression of stress biomarker genes in hive bees’ midgut. This could indicate a varying level of susceptibility, exposure or both between larvae and adult honeybees during their different developmental stages, reflected in a different detoxifying response. In addition, significant differences between plots were found in the relative expression level of several stress biomarker genes in hive bees’ midgut. However, no significant differences between plots were found in the case of larvae. In turn, we found positive correlations between pollen collection and relative gene expressions in adult workers. Moreover, PCA analysis reveals discrimination between plots for hive bees’ relative gene expression after the herbicide mixture application. Interestingly, the biomarker genes of response to pesticide exposure that distinguish between plots in the PCA are the same genes that correlate with pollen foragers’ incoming rate. On one hand, these results suggest that pollen collection and the differences in the composition of the apiaries’ surroundings might influence the detoxification response of honeybee workers. On the other hand, the effect of the herbicide mixture on larvae’s detoxifying response is not influenced by the composition of the apiaries’ surroundings.

Regarding larvae, we found significant differences concerning the relative expression of *CYP6BD1* and *CYP6AS4* in the samples after herbicide mixture application. Both *P450 cytochromes* showed a lower expression level in bees that had hatched from an egg 15 days after weed control. In accordance with this result, *CYP6AS4* was also downregulated in larvae reared in vitro and exposed to GLY [41]. Furthermore, the xenobiotic metabolism and immunity have been consistently modulated by the intake of GLY in honeybee brood in different in vitro experiments [36,42]. Additionally, honeybee colonies fed with 2,4-D reduced brood production at a concentration of 100 ppm, and eggs failed to hatch when the colony was exposed to 1000 ppm [62]. Another factor to consider is the proved disruption of gut microbiota in honeybees after GLY ingestion [63,64,65]. For instance, the presence of certain early bacterial colonizers during development, such as *Snodgrassella alvi*, can modulate phase I detoxification pathways by affecting the expression of *cytochrome P450 enzymes* [66] that are critical for pesticide degradation [67]. Once foragers return to the colony, bees can transfer contaminated food to their nestmates, by sharing food directly or through the collected resources that are eventually stored in cells [68,69]. Although we did not evaluate agrochemical residues of the studied colonies, reports confirm the presence of traces of GLY and 2,4-D in honey samples [30,31,32,33], and atrazine in pollen, wax and bees [34]. Additionally, there is evidence that herbicides and their metabolites can persist more than 20 days in water and soil [70,71,72,73]. Nevertheless, there is little information available that shows the presence of GLY in honeybee brood food, such as royal jelly and wax combs [20]. It is relatively difficult to detect GLY and its degradation metabolite using conventional methods because of their physical and chemical properties [74]. Furthermore, the possibility of the measurement of exposure biomarkers in the context of this study was low, because of the use of the good agricultural practice guidelines for agrochemicals and the herbicide mixture application with a land sprayer that led to potential residues with low concentrations that were difficult to detect [19]. However, our results of modulation in the transcription of our set of biomarkers of the detoxifying response suggest some level of chemical exposure to residual herbicides in the medium term. Nevertheless, no significant differences between plots were found for any of the genes studied in larvae.

In the case of hive bees, statistical analysis revealed significant differences between plots for the relative expression of *P450 cytochromes CYP6AS4* and *CYP9Q3*. In both cases, plot C colonies—with a higher percentage of wild flora in the surroundings—show an upregulated profile compared with plot A colonies—with a lower percentage of wild flora in the surroundings—while the apiary of plot B exhibits a similar profile to plot A colonies in the case of *CYP9Q3* and a similar profile to plot C colonies in the case of *CYP6AS4*. Among adult honeybees during the first days of their life, newly emerged bees and nurses consume pollen to develop their hypopharyngeal and mandibular glands and to produce the larval food [28]. In this sense, colonies of plot C show significantly higher pollen collection than the colonies located in the other two plots. In addition, Kendall’s rank test shows a positive and significant correlation between pollen foragers’ incoming rate and hive bees’ relative expression of *CYP6AS4*. Consistently, previous studies indicate that pollen foraging is reduced by stress and exposure to pesticide mixtures [10,11] and that pollen intake affects the level of *CYP450* transcripts [75,76]. Indeed, one of the two principal characteristics of agricultural intensification across all global landscapes, besides the increased reliance upon pesticides, is the reduction of floral diversity, resulting in a shift from heterogeneous habitats to more homogeneous ones [77]. Hence, natural and semi-natural habitats provide less disturbed foraging areas for bees which could help maintain overall biodiversity by buffering temporal variation in resources [78]. As a result, such areas can act as stabilizers, offering more prolific sources of nectar and pollen.

In general, hive bees tend to overexpress the *P450 cytochrome* detoxification genes in the second sampling moment, while the larval tendency is to downregulate these biomarker genes after herbicide mixture application. *CYP9Q3* was the only biomarker gene of response to pesticide exposure which showed a positive and significant correlation between relative expression in both hive bees and larvae, after post-emergence weed control. As colony survival depends on collective tasks, it is important to consider the exposure of bees that remain inside the hive [20]. These pre-foraging workers feed on incoming resources, as well as those stored in combs, and they perform in-hive tasks that guarantee colony care and maintenance [27]. Previous studies demonstrated that adults exposed to pesticides upregulate detoxification genes [37,39,40]. Tomé and coworkers found that multiple genes were upregulated in the newly emerged adults exposed to GLY and other agrochemicals, even though pesticide exposure occurred almost two weeks earlier during the larval stage of development [79]. On the contrary, they observed the downregulation of detoxification genes during larval exposure. In our study, we observed some similar tendencies in the regulation of detoxification pathways in larvae and young workers taken from inside colonies situated in an agricultural landscape. The downregulation of detoxification genes during the larval stage might relate to other disorders, such as low survival, poor regulation of development and various morphological disorders [79]. Additionally, enhanced catabolism and oxidative metabolism were found in honeybee larvae reared in vitro because of the sub-lethal exposure to GLY, even in the absence of observable symptoms [36].

Concerning colony activity, we found significant differences between sampling moments for total incoming rate, pollen foragers’ incoming rate and the ratio of pollen foragers to total incoming rate, with resource collection and the proportion of resources being lower after the application of herbicides. This behavior is opposite to the expected result with no negative effect of weed control. Bear in mind that in the first sampling moment before the post-emergence weed control, the three plots had only the wild flora in the foraging area, while most of the foraging area in the three plots had a high abundance of flowers in the crops and buffer zone during the second sampling. Furthermore, as stated above, colonies of plot C show a significantly higher collection of pollen and ratio of pollen foragers to total incoming rate than the colonies located in the other two plots.

Taking the hive bees’ relative expression of all genes together, after herbicide application, the PCA analysis reveals discrimination between plots as well. We would have expected that plot C colonies would be different from colonies of plot A because of the differences in the proportion of wild flora and the significant differences found in the relative expression of *CYP6AS4* and *CYP9Q3* between these plots, but the dimensions limited by PC1 and PC2 separated samples into two groups, plot B on one side and plots A and C on the other side. Interestingly, within the genes that better explain PC1 and PC2 were *CYP6AS4* and *CYP6AS2*, respectively. These are the *P450 cytochromes* that showed a positive and significant correlation with pollen foragers’ incoming rate. This indicates that pollen collection might influence the expression of these two detoxification genes. In previous studies, a significant overlap between pesticide-responsive genes and diet-responsive genes was found [40] and the expression of several members of the *CYP4* and *CYP6 cytochrome P450* families declined due to impoverished diet in nurse workers [76]. The pollen quality is poor in sunflowers (*Helianthus annuus*) and maize (*Zea mays*), with crude protein levels below the 20% level necessary to meet the basic requirements of honeybees’ nutrition [52]. Regarding sunflower, nectar is attractive to honeybees [28], and in the case of soybean (*Glycine max*), the flowers are also visited mainly for nectar collection [51]. Thus, the composition of the apiaries’ surroundings in each plot might be contributing to the differences in the detoxification response of hive bees. In this sense, an underlying interrelation between variables, involving the relative expression of all detoxification genes after post-emergence weed control, pollen collection, the sum of crop species and the species and proportion of wild flora present in the surroundings of plot B might differentiate the detoxification response in their honeybee colonies compared with the apiaries located in the other plots. On the contrary, no distinction between plots was found in PCA analysis for larvae’s relative gene expressions, after herbicide mixture application. The bulk of the pollen available to a colony is consumed by the workers [80] and to a lesser extent by larvae [28]. The high protein contents of the jellies that are fed the larvae are derived from secretions of the hypopharyngeal glands of the nurse bees [80], thus, they might consume up to 65 mg of pollen in 10 days for the development of this gland [28]. All things considered, the heterogeneity of the surroundings in the different apiaries influences the detoxification response of hive bees, suggesting that nutrition may modulate the impacts of pesticides on adult honeybees. On the one hand, *P450 cythocromes CYP6AS2*, *CYP6AS4* and *CYP9Q3* were the most modulated stress biomarker genes in hives bees. On the other hand, *CYP6AS4* and *CYP6BD1* were the most modulated stress biomarker genes in the case of larvae. Furthermore, the environmental stress after post-emergence weed control has an impact on the detoxification pathways in larvae, independently of the beehives’ surroundings. However, the energetic cost of development combined with social protection through colony-level buffering mechanisms may explain the differences in gene expression patterns between exposed larvae and adults [79].

## 5. Conclusions

In summary, these results suggest that honeybee colonies located in the agricultural landscape studied are indeed exposed to environmental stress due to the application of herbicides and the decrease in the gathering of food sources. Additionally, the detoxification response shows that larvae are more impaired than adults due to this environmental stress. The reduction of pollen collection and the exposure to agrochemicals in this disturbed landscape have an impact on honeybees’ health. The present study supports that honeybee colony surroundings in terms of the heterogeneity of resource availability may influence the detoxification response and, consequently, the impact of pesticides on adult honeybees. Regarding this matter, areas of natural habitats might contribute to preventing this trend, offering more prolific sources of nectar and pollen and protecting honeybees from drifting pesticides. To conclude, the continuity of open field assays is important to understand the global effects of agricultural management in the honeybee colony context.

## Figures and Tables

**Table 1:** Primer models. Sequence, amplified size product and melting temperature for the constitutive gene (*Rpl8*) and target genes *Abaecin*, *CYP6BD1*, *CYP9Q3*, *CYP6AS2*, *CYP6AS3*, *CYP6AS4.*

Primer	Sequence (5′ to 3′)	Product Size (MW)	Tm
*Rpl8*	F: CACACGGTGGTGGTAATCAT R: CTCGGATTCTTCCTGTACGA	114 pb	59
*Abaecin*	F: CACACTCGAGGTCTGTAGTATCT R: AATGCTGCGCATATCGTGG	111 pb	59
*CYP6BD1*	F: CTTCTGTTGCTTTTGGAATTCAAGT R: TGCATGCTGCGAGAAAATGT	106 pb	59
*CYP9Q3*	F: TTGCAAGCTCCATTCGGACA R: AACGGCCACGAATACGGTTA	130 pb	60
*CYP6AS2*	F: CGCCAGTAGACATCCCATGA R: CTGACGACATGTGTGATCAGTT	136 pb	59
*CYP6AS3*	F: GCGCGAACACTCCACCA R: CTCGTCCTCGGTACGATTTTACA	146 pb	60
*CYP6AS4*	F: TTGCGCTCTCATCTCACTCG R: AAATCGCGACAAATGCGGTT	125 pb	60

## Data Availability

The data presented in this study are available in [supplementary material here].

## Supplementary Materials

The following are available online at https://www.mdpi.com/2075-4450/12/2/163/s1, Table S1: Primer validation, Table S2: Colony activity, Table S3: Relative gene expression, Table S4: Correlation between relative gene expressions of hive bees after herbicide application, Table S5: Correlation between relative gene expressions of larvae after herbicide application, Table S6: Correlation between relative expressions of the same biomarker gene in hive bees (HB) and larvae (L), after herbicide application, Table S7: Correlation between colony activity rates and hive bees’ relative gene expressions, Table S8: Importance of components for principal component analysis (PCA), Table S9: Contribution of relative gene expressions after herbicide application to the variability in each principal component, for hive bees and larvae.
