# Supplementary material for: Evaluating the Impact of Post-Emergence Weed Control in Honeybee Colonies Located in Different Agricultural Surroundings
Source: Insects. 2021 Feb 14;12(2):163. doi: 10.3390/insects12020163 (PMC7918372; doi:10.3390/insects12020163)
Supplement: Supplementary file 1 [file insects-12-00163-s001.pdf]

insects

MDPI

Supplementary Material

Evaluating the impact of post-emergence weed control in honeybee colonies located in different agricultural surroundings

Ivana N. Macri1,2,3, Diego E. Vázquez1,2, Eduardo A. Pagano4,5, Jorge A. Zavala4,5 and Walter M. Farina1,2*

[1] Laboratorio de Insectos Sociales, Departamento de Biodiversidad y Biología Experimental, Facultad de Ciencias Exactas y Naturales, Buenos Aires, Argentina.
[2] Instituto de Fisiología, Biología Molecular y Neurociencias (IFIBYNE), CONICET-Universidad de Buenos Aires, Buenos Aires, Argentina.
[3] Instituto de Ingeniería Rural, Centro de Investigación de Agroindustria (CIA), Instituto Nacional de Tecnología Agropecuaria (INTA), Castelar, Buenos Aires, Argentina.
[4] Cátedra de Bioquímica, Facultad de Agronomía, Universidad de Buenos Aires, Buenos Aires, Argentina.
[5] Instituto de Investigaciones en Biociencias Agrícolas y Ambientales, (INBA), CONICET-Universidad de Buenos Aires, Buenos Aires, Argentina.
* Correspondence: walter@fbmc.fcen.uba.ar

Table S1. Primer validation. Efficiency, slope and R^2 values for housekeeping and target genes obtained in validation relative standard curve. Models were made up using Primer 3 and BLAST software

	Hive bees		
	**Gene**	**Efficiency (%)**	**R^2**
TARGETS	*ABAECIN*	100.412	0.987
	CYP6BD1	109.38	0.959
	CYP6AS2	107.558	0.836
	CYP6AS3	98.541	0.923
	CYP6AS4	106,517	0.836
	CYP9Q3	102.235	0.966
HOUSEKEEPING	*RPL8*	104.382	0.754
	**Larvae**		
	**Gene**	**Efficiency**	**R^2**
TARGETS	*ABAECIN*	104.319	0.987
	CYP6BD1	106,031	0.981
	CYP6AS2	109.929	0.876
	CYP6AS3	100.650	0.975
	CYP6AS4	109.188	0.929
	CYP9Q3	99.05	0.876
HOUSEKEEPING	*RPL8*	92.918	0.929

Table S2. Colony activity. Statistics of Wilcoxon paired test for comparisons between applications moments. Statistics of Friedman rank sum test for comparisons between plots. Significant differences in bold.

**Total incoming rate**				
**Comparisons**	**Test**	**Statistic**	**95 percent confidence interval**	**p value**
Between pre and post herbicide application	Wilcoxon	V=289, estimate=40.898	28 - 52.5	**<0.001**
Between plots	Friedman	$\chi2$=0.143, df=2		0.931
**Pollen foragers' incoming rate**				
**Comparisons**	**Test**	**Statistic**		**p value**
Between pre and post herbicide application	Wilcoxon	V=253, estimate=11.999	9 - 15	**<0.001**
Between plots	Friedman	$\chi2$=8.044, df=2		**0.018**
Plot A - Plot B	Conover post hoc			**<0.001**
Plot A - Plot C	Conover post hoc			0.24
Plot B - Plot C	Conover post hoc			**<0.001**
**Ratio Pollen/Total incoming rate**				
**Comparisons**	**Test**	**Statistic**		**p value**
Between pre and post herbicide application	Wilcoxon	V=280, estimate=0.133	0.087 - 0.181	**<0.001**
Between plots	Friedman	$\chi2$=9.805, df=2		**0.007**
Plot A - Plot B	Conover post hoc			**<0.001**
Plot A - Plot C	Conover post hoc			0.31
Plot B - Plot C	Conover post hoc			**<0.001**

Table S3. Relative gene expression. Statistics of Wilcoxon paired test for comparisons between herbicides applications moments. Statistics of Friedman rank sum test for comparisons between plots. Significant differences in bold.

**Hive bees**					
**Gene**	**Comparisons**	**Test**	**Statistic**	**95 percent confidence interval**	**p value**
ABAECIN	Between pre and post herbicide application	Wilcoxon	V=36, estimate= -0.105	-2.537 - 1.550	0.814
	Between plots	Friedman	χ2=0, df=2		1
CYP6BD1	Between pre and post herbicide application	Wilcoxon	V=24, estimate= -0.194	-2.681 - 0.447	0.424
	Between plots	Friedman	χ2=4.75, df=2		0.093
CYP6AS2	Between pre and post herbicide application	Wilcoxon	V=56, estimate=0.862	-0.835 - 3.286	0.204
	Between plots	Friedman	χ2=1.75, df=2		0.417
CYP6AS3	Between pre and post herbicide application	Wilcoxon	V=37, estimate= -0.194	-1.971 - 9.829	0.875
	Between plots	Friedman	χ2=1, df=2		0.606
CYP6AS4	Between pre and post herbicide application	Wilcoxon	V=29, estimate= -0.559	-6.344 - 2.000	0.470
	Between plots	Friedman	χ2=9.25, df=2		**0.01**
	Plot A - Plot B	Conover post hoc			**<0.001**
	Plot A - Plot C	Conover post hoc			**<0.001**
	Plot B - Plot C	Conover post hoc			0.618
CYP9Q3	Between pre and post herbicide application	Wilcoxon	V=20, estimate= -0.033	-1.522 - 3.083	0.248
	Between plots	Friedman	χ2=9.75, df=2		**0.008**
	Plot A - Plot B	Conover post hoc			**<0.001**
	Plot A - Plot C	Conover post hoc			0.140
	Plot B - Plot C	Conover post hoc			**<0.001**
**Larvae**					
**Gene**	**Comparisons**	**Test**	**Statistic**	**95 percent confidence interval**	**p value**
ABAECIN	Between pre and post herbicide application	Wilcoxon	V=39, estimate= -0.075	-2.267 - 1.731	1
	Between plots	Friedman	χ2=0.25, df=2		0.882
CYP6BD1	Between pre and post herbicide application	Wilcoxon	V=66, estimate=1.451	0.017 - 5.939	**0.034**
	Between plots	Friedman	χ2=0.25, df=2		0.882

CYP6AS2	Between pre and post herbicide application	Wilcoxon	V=38, estimate= -0.107	-2.674 - 2.051	0.970
	Between plots	Friedman	χ2=0.25, df=2		0.883
CYP6AS3	Between pre and post herbicide application	Wilcoxon	V=63, estimate=1.856	-0.212 - 5.964	0.064
	Between plots	Friedman	χ2=0.25, df=2		0.882
CYP6AS4	Between pre and post herbicide application	Wilcoxon	V=70, estimate=0.979	0.177 - 2.920	**0.012**
	Between plots	Friedman	χ2=3.161, df=2		0.206
CYP9Q3	Between pre and post herbicide application	Wilcoxon	V=61, estimate=1.019	-0.366 - 2.380	0.092
	Between plots	Friedman	χ2=1, df=2		0.606

Table S4. Correlation between relative gene expressions of hive bees after herbicide application. Tau, z values and p values obtained from Kendall's rank correlation test. Significant differences in bold.

Correlated genes	tau	z value	p value
ABAECIN-CYP6BD1	0.292	1.309	0.191
ABAECIN-CYP6AS2	0.321	1.443	0.149
ABAECIN-CYP6AS3	-0.076	-0.344	0.731
ABAECIN-CYP6AS4	0.015	0.069	0.945
ABAECIN-CYP9Q3	0.107	0.481	0.630
CYP6BD1 - CYP6AS2	**0.779**	**3.506**	**<0.001**
CYP6BD1 - CYP6AS3	0.259	1.169	0.2426
CYP6BD1 - CYP6AS4	**0.657**	**2.956**	**0.003**
CYP6BD1 - CYP9Q3	**0.748**	**3.368**	**<0.001**
CYP6AS2- CYP6AS3	0.303	1.433	0.197
CYP6AS2- CYP6AS4	**0.515**	**2.501**	**0.021**
CYP6AS2- CYP9Q3	**0.667**	**3.155**	**0.002**
CYP6AS3- CYP6AS4	0	0.033	1
CYP6AS3-CYP9Q3	0.152	0.538	0.5452
CYP6AS4 - CYP9Q3	**0.848**	**4.132**	**<0.001**

Table S5. Correlation between relative gene expressions of larvae after herbicide application. Tau, z values and p values obtained from Kendall's rank correlation test. Significant differences in bold.

Correlated genes	tau	z value	p value
ABAECIN-CYP6BD1	0.242	1.041	0.311
ABAECIN-CYP6AS2	-0.168	-0.756	0.450
ABAECIN-CYP6AS3	0.273	1.240	0.250
ABAECIN-CYP6AS4	0.164	0.714	0.475
ABAECIN-CYP9Q3	0.382	1.718	0.086
CYP6BD1 - CYP6AS2	0.076	0.344	0.731
CYP6BD1 - CYP6AS3	0.424	1.847	0.063
CYP6BD1 - CYP6AS4	0.428	1.857	0.063
CYP6BD1 - CYP9Q3	0.290	1.306	0.191
CYP6AS2- CYP6AS3	0.351	1.581	0.113
CYP6AS2- CYP6AS4	0.249	1.074	0.283
CYP6AS2- CYP9Q3	-0.246	-1.102	0.270
CYP6AS3- CYP6AS4	**0.461**	**2**	**0.046**
CYP6AS3-CYP9Q3	0.015	0.069	0.945
CYP6AS4 - CYP9Q3	0.348	1.503	0.133

Table S6. Correlation between relative expressions of the same biomarker gene in hive bees (HB) and larvae (L), after herbicide application. Tau, z values and p values obtained from Kendall's rank correlation test. Significant differences in bold.

Correlated genes	tau	z value	p value
ABAECIN HB – ABAECIN L	0.198	0.894	0.371
CYP6BD1 HB – CYP6BD1 L	0.046	0.206	0.837
CYP6AS2 HB – CYP6AS2 L	-0.321	-1.443	0.149
CYP6AS3 HB – CYP6AS3 L	-0.091	0.430	0.737
CYP6AS4 HB – CYP6AS4 L	0.132	0.571	0.568
CYP9Q3 HB – CYP9Q3 L	**0.504**	**2.268**	**0.023**

Table S7. Correlation between colony activity rates and hive bees 'relative gene expressions. Tau, z values and p values obtained from Kendall's rank correlation. Significant differences in bold.

Correlated variables	tau	z value	p value
Total incoming rate- *ABAECIN*	0.099	0.671	0.502
Total incoming rate-*CYP9Q3*	-0.153	-1.024	0.306
Total incoming rate-*CYP6BD1*	-0.073	0.497	0.619
Total incoming rate-*CYP6AS2*	0.080	0.546	0.585
Total incoming rate-*CYP6AS3*	0.077	0.522	0.602
Total incoming rate-*CYP6AS4*	0.077	0.522	0.602
Pollen foragers' incoming rate- *ABAECIN*	0.273	1.759	0.078
Pollen foragers' incoming rate- *CYP9Q3*	0.037	0.234	0.815
Pollen foragers' incoming rate-*CYP6BD1*	-0.004	-0.026	0.979
**Pollen foragers' incoming rate- *CYP6AS2***	**0.328**	**2.121**	**0.034**
Pollen foragers' incoming rate- *CYP6AS3*	0.124	0.802	0.422
**Pollen foragers' incoming rate- *CYP6AS4***	**0.349**	**2.251**	**0.024**
**Ratio Pollen/Total incoming rate- *ABAECIN***	**0.369**	**2.402**	**0.016**
Ratio Pollen/Total incoming rate- *CYP9Q3*	0.057	0.363	0.716
Ratio Pollen/Total incoming rate- *CYP6BD1*	-0.008	0.052	0.959
Ratio Pollen/Total incoming rate- *CYP6AS2*	0.242	1.575	0.115
Ratio Pollen/Total incoming rate- *CYP6AS3*	0.079	0.516	0.605
Ratio Pollen/Total incoming rate- *CYP6AS4*	0.206	1.342	0.179

Table S8. Importance of components for principal component analysis (PCA). Standard deviation, proportion of variance and cumulative proportion for each principal component.

**Hive bees**						
	**PC1**	**PC2**	**PC3**	**PC4**	**PC5**	**PC6**
Standard deviation	1.681	1.093	1.055	0.894	0.245	0.089
Proportion of Variance	0.471	0.199	0.185	0.133	0.010	0.001
Cumulative Proportion	0.471	0.670	0.856	0.989	0.999	1.000
**Larvae**						
	**PC1**	**PC2**	**PC3**	**PC4**	**PC5**	**PC6**
Standard deviation	1.516	1.356	0.998	0.808	0.415	0.204
Proportion of Variance	0.383	0.306	0.166	0.109	0.029	0.007
Cumulative Proportion	0.383	0.689	0.855	0.964	0.993	1.000

Table S9. Contribution of relative gene expressions after herbicide application for the variability in each principal component, for hive bees and larvae.

**Hive bees**						
	**PC1**	**PC2**	**PC3**	**PC4**	**PC5**	**PC6**
ABAECIN	0.096	-0.077	**0.760**	-0.636	0.034	0.020
CYP6BD1	**-0.559**	-0.221	-0.091	-0.128	0.777	-0.103
CYP6AS2	-0.179	**-0.795**	-0.271	-0.278	-0.430	0.025
CYP6AS3	0.138	0.394	-0.571	-0.706	0.032	0.022
CYP6AS4	**-0.567**	0.255	0.075	-0.016	-0.230	0.745
CYP9Q3	**-0.553**	0.305	0.092	-0.051	-0.396	-0.658
Larvae						
	**PC1**	**PC2**	**PC3**	**PC4**	**PC5**	**PC6**
ABAECIN	0.069	-0.102	**0.982**	-0.116	-0.070	0.054
CYP6BD1	0.148	**0.688**	0.031	0.027	-0.664	-0.249
CYP6AS2	-0.536	0.044	-0.080	-0.690	-0.257	0.403
CYP6AS3	-0.521	0.136	0.102	0.701	-0.108	0.443
CYP6AS4	**-0.624**	0.172	0.117	-0.033	0.302	-0.689
CYP9Q3	0.157	**0.683**	0.071	-0.132	0.620	0.319
